# The PARP Enzyme Family and the Hallmarks of Cancer Part 1. Cell Intrinsic Hallmarks

**DOI:** 10.3390/cancers13092042

**Published:** 2021-04-23

**Authors:** Máté A. Demény, László Virág

**Affiliations:** 1 Department of Medical Chemistry, Faculty of Medicine, University of Debrecen, 4032 Debrecen, Hungary; 2 MTA-DE Cell Biology and Signaling Research Group, University of Debrecen, 4032 Debrecen, Hungary

**Keywords:** hallmarks of cancer, poly (ADP-ribose) polymerase, oncogenes, tumor suppressors, cell death, replicative immortality, metabolic reprogramming

## Abstract

**Simple Summary:**

Poly (ADP-ribose) polymerase (PARP) proteins regulate DNA damage correction, replication, and gene transcription. By controlling pivotal aspects of these processes, PARPs are heavily implicated in cancer development. Inhibitors of PARPs, approved for cancer chemotherapy a few years ago, have achieved great success against tumors of the breast and ovary carrying mutations in the BRCA1/2 genes. The spectrum of the inhibitors is avidly sought to be extended to tumors with different genetic backgrounds and cancers of other origins. This pursuit requires thorough apprehension of PARP-dependent processes affecting cancer development. The hallmarks of cancer are acquired by defining capabilities that differentiate cancer cells from their normal counterparts. Here, in two joint papers, we walk through the connections between these cancer traits and PARP functions. The present review focuses on how PARPs affect the features of cancer that can be attributed to cell-intrinsic changes increasing proliferative potential and survival capabilities. In a kindred paper, we explore the PARP association of cancer hallmarks that derive from tissue-level reorganization in tumors and intercellular interactions of cancer cells.

**Abstract:**

The 17-member poly (ADP-ribose) polymerase enzyme family, also known as the ADP-ribosyl transferase diphtheria toxin-like (ARTD) enzyme family, contains DNA damage-responsive and nonresponsive members. Only PARP1, 2, 5a, and 5b are capable of modifying their targets with poly ADP-ribose (PAR) polymers; the other PARP family members function as mono-ADP-ribosyl transferases. In the last decade, PARP1 has taken center stage in oncology treatments. New PARP inhibitors (PARPi) have been introduced for the targeted treatment of breast cancer 1 or 2 (BRCA1/2)-deficient ovarian and breast cancers, and this novel therapy represents the prototype of the synthetic lethality paradigm. Much less attention has been paid to other PARPs and their potential roles in cancer biology. In this review, we summarize the roles played by all PARP enzyme family members in six intrinsic hallmarks of cancer: uncontrolled proliferation, evasion of growth suppressors, cell death resistance, genome instability, reprogrammed energy metabolism, and escape from replicative senescence. In a companion paper, we will discuss the roles of PARP enzymes in cancer hallmarks related to cancer-host interactions, including angiogenesis, invasion and metastasis, evasion of the anticancer immune response, and tumor-promoting inflammation. While PARP1 is clearly involved in all ten cancer hallmarks, an increasing body of evidence supports the role of other PARPs in modifying these cancer hallmarks (e.g., PARP5a and 5b in replicative immortality and PARP2 in cancer metabolism). We also highlight controversies, open questions, and discuss prospects of recent developments related to the wide range of roles played by PARPs in cancer biology. Some of the summarized findings may explain resistance to PARPi therapy or highlight novel biological roles of PARPs that can be therapeutically exploited in novel anticancer treatment paradigms.

## 1. Introduction

The PARP enzyme family, consisting of 17 members (the original publication reported 18 PARP superfamily members but later tankyrase 3 turned out to be a shorter splice variant of tankyrase 2 (TNKS2)), was first described in 2004 as a group of proteins sharing the conserved PARP signature sequence [1] (Supplementary Figure S1). ADP-ribosyl transferase (ART) enzymes cleave NAD^+^ to ADP-ribose and nicotinamide, and attach the former cleavage product to serine, glutamate, aspartate, arginine, lysine, or cysteine residues of proteins or to DNA/RNA ends [2,3] (Supplementary Figure S2). Some small chemical groups, such as acetate or phosphate, may also be ADP-ribosylated by sirtuins and the KptA/Tpt1-like RNA phosphotransferase enzymes, respectively [4,5]. ART enzymes showing similarity to the cholera toxin are dubbed ARTC, whereas the diphtheria toxin-like ARTs are known as ARTDs [6]. Most ARTs modify their targets with a single ADP-ribose moiety, which is referred to as mono (ADP-ribosyl)ation (MARylation). Some ARTs, however, can elongate the first protein-bound unit to form longer and sometimes branched poly (ADP-ribose) (PAR) polymers. From the established mammalian PARP family, only six members (PARP1-6) were later validated as bona fide PARP enzymes, whereas the rest of the family members were recognized to function as mono-ADP-ribosyl transferases (MART). This controversy later led to a new classification, equally pervasive in the literature, that catalogs members of the original PARP family as ARTDs [6] (Supplementary Figure S1).

MARylated and PARylated proteins are specifically recognized by proteins possessing ADP-ribose-binding motifs (e.g., macrodomain, PAR-binding motif, PAR-binding zinc finger, WWE domain, and RNA and DNA binding motifs) [5]. PARylation and MARylation are reversed by poly (ADP-ribose) glycohydrolase (PARG), ADP-ribosyl hydrolase (ARH) family members (ARH1, ARH3), terminal ADP-ribose protein glycohydrolase 1 (TARG1), MacroD1 and MacroD2 enzymes, and some phosphodiesterases [7]. The biological functions of PARPs can also be mediated by protein–protein interactions or by modulation of cellular NAD levels [8].

ADP-ribosylation plays a role in DNA repair, replication, transcription, telomere dynamics, and metabolism [5]. Through these molecular events, ADP-ribosylation regulates cell proliferation, differentiation, cell death, and immunity, implicating PARPs in cancer development [9]. Targeting PARPs with inhibitors has become part of oncological practice in recent years. As of now, four PARPis are approved for clinical use by the US Food and Drug Administration (FDA). Olaparib, niraparib, and rucaparib are approved for the treatment of high-grade serous ovarian cancer, olaparib and talazoparib are approved for the treatment of metastatic breast cancer, and olaparib is approved for the treatment of germline BRCA-mutated (gBRCAm) metastatic pancreatic adenocarcinoma (Supplementary Table S1). This treatment modality takes advantage of the dependence of homologous recombination-deficient cancer cells on PARP1 and PARP2 for the repair of endogenous DNA lesions [10,11,12]. 

Hanahan and Weinberg described the quintessential traits of cancer that distinguish malignant tissues from their normal counterparts in two seminal papers in 2000 and 2011 [13,14]. These two reviews consolidated the modern concept of cancer by drawing on the somatic mutation theory (SMT), which identified cancer as a genetic disease caused by inherited genetic alterations and exogenously induced mutations, and incorporated the idea that cancer was an evolutionary process in which a genetically heterogeneous population of cells enabled by the sequential acquisition of mutations vied for proliferative advantage, nutrients, and the evasion of the immune system [15,16]. The first set of hallmarks was a testimony to the confidence and optimism that most of the pathomechanistic traits of cancer had been uncovered, and offered hope of an imminent success in combating the disease. The second paper, published a decade later, incorporated amendments to the original set of hallmarks confronted with lasting therapeutic challenges and emerging alternative pathomechanistic hypotheses. It was recognized that cancer was a systems disease and a tumor was no lump of hyperproliferative cells, but more like a complex organ that could not be adequately understood in terms of features autonomous only to the cancerous cells. Increasing evidence supported that non-genetic/epigenetic changes in the perturbed expression of regulatory factors contributed to the development of cancer and its resistance to therapy [17]. The new hallmarks described communication between the cancer tissue and the host organism, exemplified by tumor vascularization, invasion and metastasis, cancer-associated inflammation, and evasion of immune attacks on cancer. This set of hallmarks is discussed in detail in the companion paper (part 2) of this review [18].

Ever since their publication, the hallmarks of cancer have been a useful framework for many investigators working in diverse fields of cell biology to connect their particular cellular processes of interest to features of the disease. Although PARPs and PARylation are closely intertwined with the development, spreading, and treatment of cancer, no comprehensive review has been published, to our knowledge, that systematically interrogates all members of the PARP family in relationship to cancer hallmarks. The aim of this review is to focus on how the pleiotropic normal functions of PARPs contribute to or impede the molecular circuitries relevant to the transformation, immortality, growth, death resistance, and metabolic adaptation of cancer cells. 

Currently, the strongest connection between PARylation and cancer biology is represented by a novel anticancer therapy; PARP inhibitors are approved for the treatment of BRCA-deficient ovarian and breast cancers. The mechanism and therapeutic spectrum of PARPi therapy and its potential extension to chemo- and radiosensitization have been the subject of numerous excellent reviews [10,12,19]. Thus, these issues will not be covered here. We focus, instead, on the multilevel overlap between the wide-ranging biological functions of PARP/ARTD enzymes and the hallmarks of cancer (HoC). Since the HoC highlight the most essential traits of cancer, pinpointing the involvement of PARP enzymes (especially the least known and mostly neglected PARP family members) in the modulation of HoC is important. The PARP family is comprised of 17 members and single PARP proteins can be involved in several of these circuits; at times, PARPs have antagonistic roles in tumorigenesis through their involvement in different pathways. Furthermore, none of the clinically used PARPi drugs specifically target a single PARP enzyme [20]. Therefore, some of the potential beneficial and/or unwanted effects of PARPi drugs may be due to the inhibition of “minor” PARP family members. A better understanding of the plethora of effects that PARP functions have on the various aspects of cancer development is necessary for the refined utilization of PARPis and the prevention of potential unwanted consequences. The involvement of PARPs in the analogous pathways in healthy cells needs to be given ample consideration to minimize side effects. 

## 2. PARPs in Hallmark Cancer Traits 

### 2.1. Sustaining Proliferative Signaling and Evading Growth Suppressors

**Figure 1:**
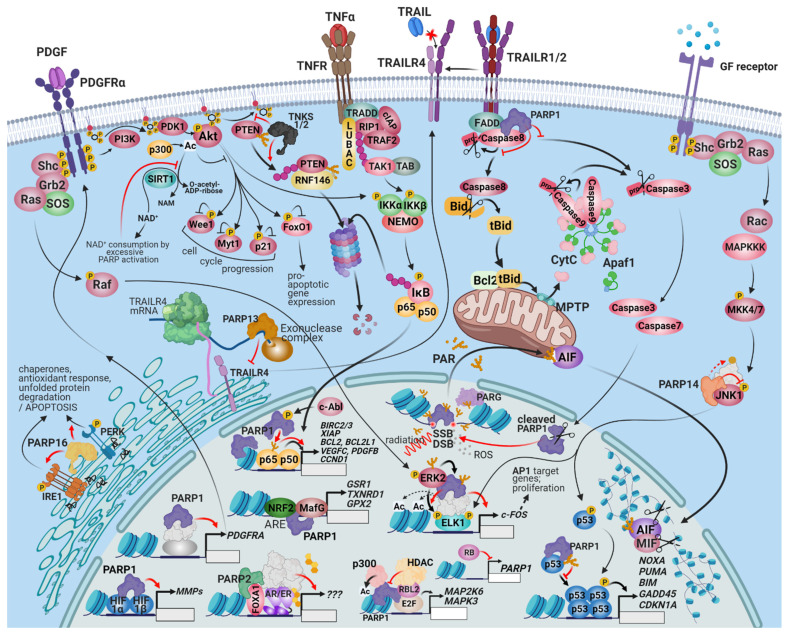
The connection between PARPs, prosurvival signaling, and cell death resistance. PARP1 and 2 are important cell death resistance and prosurvival factors because of their dominant role in multiple DNA repair pathways, fork reactivation, and the execution of mitosis. PARPis target these functions. Interactions between the PARP family and cell survival regulation, however, extends beyond the modulation of specific gene transcription and signaling events by PARP proteins. *PARP-1* is involved in the upregulation of PDGFRα, contributing to autocrine/paracrine survival growth factor signaling in neuroblastomas. *PARP-2* is a coactivator of nuclear hormone receptors and contributes to the growth of hormone-dependent prostate and breast carcinomas. The PARPs regulate several MAPK pathways as well. The interaction of *PARP-1* with ERK facilitates the phosphorylation of ELK and other ERK-dependent stimulatory chromatin alterations on the promoters of cell-survival inducing genes. *PARP-14* is an effector of JNK1/2-dependent prosurvival signaling in various cancers. *PARP-1* is a negative regulator of the prosurvival RTK-PI3K-Akt pathway; the consumption of available cytoplasmic NAD^+^ by *PARP-1* prevents the deacetylation of Akt by SIRT1, which may be important for the tissue-saving effect of PARPis after ischemic damage. TNKS1/2, however, support Akt signaling by PARylating and promoting the degradation of PTEN. The interconnection of the PI3K-Akt pathway with the NRF2 and HIF pathways makes the outcome of PARP activation/inhibition controversial and potentially cell-type specific. During substantial genotoxic stress, PAR chains produced by *PARP-1* and *2* at DNA damage sites and released by PARG translocate into the mitochondria and induce parthanatos, a programmed necrosis. One of the primary mediators of parthanatos is AIF, a liberated mitochondrial factor that induces the macrophage migration inhibitory factor (MIF) to cleave the nuclear DNA into 20–50 kb fragments. A distinct cytoplasmic subset of *PARP-1* is recruited into the DISC and is involved in interference with the extrinsic apoptosis pathway and in the sustained activation of Src survival signaling. The role of the rest of the PARPs in cell death signaling is also multimodal. *PARP-13* facilitates the degradation of the mRNA of the decoy receptor, TRAILR4, restoring TRAIL sensitivity and facilitating apoptosis. PARP16, an ER-localized transmembrane protein, PARylates PERK and IRE-1α and facilitates their role in the ER-stress response, which can be adaptive or lead to apoptosis. Similarly, the outcome of p53 activation can range from cell survival to programmed cell death; consequently, the net result of p53–PARP1 interaction is expected to be cell-type and context-dependent. PARP1 recruits p300 to a set of E2F-dependent promoters, which results in the expression of proliferation-enhancing target genes. Retinoblastoma-like protein 2 (RBL2) counteracts the opening of the chromatin at these promoters. The retinoblastoma protein inhibits the expression of PARP1.

One of the most widely known HoC is uncontrolled proliferation driven by upregulated growth factor signaling and inactivation of growth suppressor mechanisms [13,14]. The most common mechanisms underlying the hyperresponsiveness of cancer cells to mitogenic signaling include increased expression of growth factor receptors (GFR) and their ligands (e.g., epidermal GFR (EGFR) in nonsmall cell lung cancers) and constitutively active GFR signaling pathways (e.g., mutated b-Raf driving MAP kinase cascades in melanoma or mutated PI3 kinase stimulating Akt kinase in various tumors) (Figure 1). As for the inactivation of growth suppressor mechanisms, mutations in the tumor suppressor p53 and retinoblastoma proteins (pRb) are the most plausible examples. Moreover, defects in proteins counteracting proliferation signaling (e.g., the phosphatase and tensin homolog (PTEN) phosphatase reversing the PI3 kinase pathway or inactivation of the GTPase activity of Ras proteins) and disruption of contact inhibition signaling (e.g., via liver kinase B1 (LKB1) cell polarity regulator proteins or the *neurofibromin 2* (*NF2*) gene product Merlin) should also be listed here [13,14]. 

**Figure 2:**
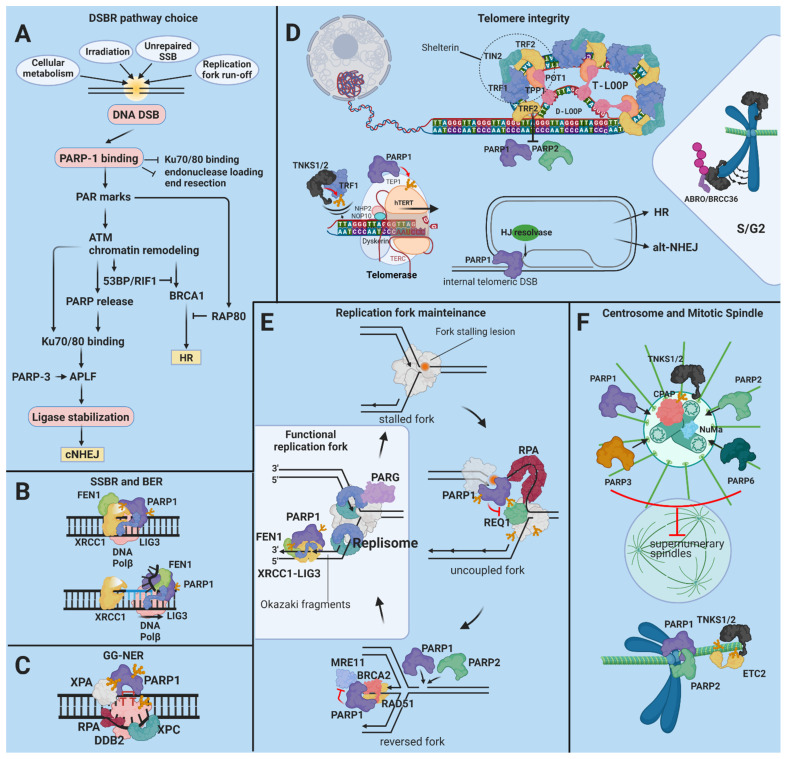
The role of the PARPs in protection from genetic instability. (**A**) Milliseconds after a DSB, *PARP-1* secures an open pathway choice via protection of the HR pathway from interference by the Ku70/80 complex and by recruiting but temporarily antagonizing MRE11-containing exonuclease complexes [140,141,142,143,144]. Chromatin remodelers recruited to PAR marks open the chromatin for the repair factors. Consecutively, *PARP-1* facilitates the recruitment of the 53BP-RIF1 complex and NONO, suppressors of DNA resection, which enhance NHEJ. At the same time, *PARP-1* stimulates the interaction of BRCA1, a governing factor of HR, with the inhibitory RAP80 complex [145,146,147]. *PARP-3* enhances the retention of the XRCC4/DNA ligase IV complex at the DSB site before the completion of NHEJ [149,150]. *PARP-2* facilitates DNA resection, leading to a different pathway choice in favor of HR [141,151] TNKS1/2 facilitate the arrival of BRCA1 by recruiting and promoting chromatin remodeling through MDC1. TNKS1/2 also stabilize the BRCA1 complex through the recruitment of MERIT40 [152]. (**B**) PARylated *PARP-1* or other proteins serve as a scaffold for XRCC1, DNA Polβ, FEN1, and PNKP during BER. *PARP-1* directly stimulates strand replacement synthesis by Polβ and 5′-flap cleavage by FEN1 during LP-BER [165]. (**C**) *PARP-1* directs DDB2 and XPC to the UV-induced lesions. Tripartite interactions between PARP1, XPA, and RPA answer for the spatio-temporal fine-tuning of the NER complex assembly. (**D**) TNKS1 is indispensable for the regulation of sister telomere resolution during the cell cycle. TNKS1 associates with the telomere and is K63-ubiquitylated in late S/G2 by RNF8, reinforcing telomere cohesion. (**E**) At stalled replication forks, PARP1 delays the activation of the DNA helicase RECQ1, postponing fork restart [172]. *PARP-1*, *PARP-2*, and BRCA2 cooperate to stabilize RAD51, facilitating fork reversal. Fork reversal is necessary for clearing the way for lesion repair [173,174]. PARP1 also recruits and, together with BRCA2, fine-tunes the exonuclease activity of MRE11, which is required for DNA resection at the reversed fork before it can be restarted [175,176]. (**F**) *PARP-1*, *PARP-3*, *PARP-6*, and TNKS1/2 associate with the centrosome and control its amplification, preventing the formation of supernumerary spindles. The cytokinesis regulating factor ETC2 is recruited to spindle microtubules PARylated by TNKS1 during metaphase [177]. *PARP-1* and *PARP-2* interact with CENP-A, CENP-B, and the mitotic checkpoint complex (MCC) at active centromeres to regulate the metaphase-anaphase transition [178,179].

*PARP-1* is closely intertwined with both proliferation regulatory circuitries and their suppressor mechanisms. The closest relationship between *PARP-1* and proliferation exists in the DNA replication process itself, as evidenced by the destabilization of replication forks in PARPi-treated cancer cells. *PARP-1* interacts with the multiprotein DNA replication complex (MRC), also called the DNA synthesome, and PARylates at least 15 of its ca. 40 components [21]. *PARP-1* may also play a role in the assembly of the active DNA synthesome [21] (Figure 2E). *PARP-1* controls the expression of E2 promoter binding factor 1 (E2F-1) by increasing its promoter activity in the early S phase [22]. DNA replication occurs during the S phase of the cell cycle, but the role of PARPs goes beyond the S phase and affects other critical mitotic events, such as mitotic spindle assembly and telomere elongation (Figure 2D,F). PARP1, PARP3, and tankyrase-1 all associate with and regulate the function of the centrosome [23,24,25]. PARP1 PARylates centrosomal p53 (see below) and other centrosomal proteins. When PARP activity is inhibited or the PARP1 gene is inactivated, centrosome hyperproliferation occurs [26] (Figure 2F).

Several tumor-driving growth factors are regulated by PARP1. For example, platelet-derived growth factor receptor α (PDGFRα) stimulates the growth of gliomas [27] and several other cancer types. *PARP-1* (even in the enzymatically inactive form) is a transcriptional activator of PDGFRα in neuronal stem cells [28] (Figure 1). Whether *PARP-1* plays a similar role in gliomas and other PDGFRα-driven tumors is unclear. Furthermore, PARP2 is a coactivator of the androgen receptor and contributes to the growth of androgen-dependent prostate cancer [29]. The underlying mechanism involves the interaction of PARP2 with forkhead box protein A1 (FOXA1), a pioneer transcription factor (TF) that mediates early events of transcription (Figure 1). FOXA1 interacts with and is required for the action of the androgen receptor. Since FOXA1 plays similar roles in the transactivation of other nuclear receptors (e.g., estrogen receptor) [30], this finding may be relevant to estrogen receptor-positive breast cancers. When prostate cancers become unresponsive to androgens, upregulation of insulin-like growth factor 1 (IGF-1) is often detected [31]. Similarly, the development of resistance to GSK3 inhibitor treatment in breast cancers is often caused by the upregulation of IGF-1. Thus, the interactions between PARPs and epidermal growth factor (EGF) signaling are highly relevant. PARPis synergize with the IGF-1R kinase inhibitors in BRCA1 mutant (HR-deficient) ovarian cancer cells [31].

The proliferation regulatory role of PARP1 reaches beyond the level of growth factor (GF)-receptor activation. PARP1 also affects downstream signaling events. For example, PARP1 interacts with extracellular signal regulated kinase-2 (ERK-2), leading to a DNA-independent ERK-2-mediated PARP1 activation that amplifies Erk-2-related epigenetic alterations [32] (Figure 1). The upregulation of the PI3K-Akt pathway by PARP inhibition also fits this trend, as discussed in Section 3. 

The close connection between PARP1 and the tumor suppressor protein p53 is also of great interest to those who study the complex role of PARP1 in cancer promotion or suppression. The central orchestrator of the stress response is p53 [33]. The abundance of p53 is regulated by the E3 ubiquitin ligase Mdm2, and p53 activity is controlled by a myriad of post-translational modifications (PTM), including phosphorylation, acetylation, methylation, and sumoylation. The PTMs lead to conformation changes in p53 and interactions with partner proteins in a PTM profile-dependent manner [34]. Acting as a transcription factor, p53 controls the expression of ca. 100 genes. Activation of p53 regulates DNA damage response, proliferation, senescence, and cell death. Mutation of p53, as observed in many cancers, is a cancer driving mechanism and may confer new oncogenic traits to cancer [35].

The functions of PARP1 and p53 are intertwined at many levels (Figure 1). PARP1 PARylates p53 [36], and PARylation acts as a PTM code determining the outcome of p53 activation. Indeed, PARP1 modifies the effectiveness of the p53-mediated DNA damage response [37]; PARylated p53 is unable to bind to its consensus sequence [36]. *PARP-1* is also a critical regulator of p53-mediated p21CIP1 induction and G1-arrest in MCF-7 and BJ/TERT cells. Inhibition of *PARP-1* in these cells suppresses p53 activation in response to ionizing radiation-induced DNA damage [38]. *PARP-1* stabilizes p53 and retains the mutant p53, which is tumorigenic due to the loss of nuclear localization [39]. The nuclear export of p53 is blocked by PARP-1-mediated PARylation that inhibits the p53 interaction with the nuclear export receptor Crm1. Thus, *PARP-1* promotes the nuclear accumulation of p53, where it exerts its transcriptional function [40]. Moreover, p53 status affects cancer cell sensitivity to PARPi therapy [41]. Thus, the functional interplay between PARP1 and p53 is bidirectional and its consequences largely depend on the cell injury model. 

The tumor suppressor protein pRb is mutated in various forms of cancer [42]. The primary effects of pRb are related to cell cycle control at the level of G1 to S phase. The E2F family of transcription factors is inhibited by pRb; in the active state, E2F proteins drive the G1 to S transition via induction of cyclin E and A expressions. Cyclin E and A are activators of Cdk2 [42]. The pRb-mediated E2F inhibition involves the recruitment of histone deacetylases and chromatin compaction. The pRb activity is controlled mainly by phosphorylation via Cdk enzymes and dephosphorylation by protein phosphatase 1 (PP1). One of the first observations linking PARP1 to pRb came from the Smulson laboratory. Smulson showed that PARP1 knockout fibroblasts failed to progress to the S phase due to severely reduced E2F promoter activity after release from serum-deprived conditions [43]. The crosstalk between PARP1 and pRb is bidirectional as PARP1 expression is downregulated by pRb in the presence of adenoviral early antigen E1A [44]. Moreover, in pRb-mutated cancer cells, where entry into S phase is unimpeded, PARPi or PARP1 knockdown sensitized cells to DNA-damaging chemotherapeutics; thus, caution should be used when treating pRb mutated cancers with PARPi therapy [45]. However, the role of pRb in genotoxic stress appears to be more direct; the protein is recruited to and assists in homologous recombination repair of DNA double-strand breaks. Furthermore, some pRb-dependent genes are co-regulated by PARP1 via the histone acetyltransferase p300 [46]. Thus, the interplay between PARP1 and pRb is multidimensional and covers not only the regulation of cancer cell proliferation, but also the sensitivity to chemotherapeutics and, theoretically, also to radiation.

Senescence is a special form of cell cycle arrest representing an escape mechanism for cancer cells [47]. Both p53 and pRb are mediators of the senescence process, which leads cells to a viable, actively metabolic but nonproliferative state [47]. Moreover, an increasing body of evidence suggests that senescent cells can revert to a nonsenescent phenotype which, in cancer, is manifested by renewed cancer growth [48]. In breast, ovarian cancer cells, and irradiated colon cancer cells, PARPi induces senescence [49,50,51]. Furthermore, senolytic drugs display synthetic lethality with PARPi, as demonstrated in ovarian and breast cancer models [52]. These findings suggest that PARylation negatively regulates the senescence pathway and senescence inhibition may contribute to the chemo- or radiosensitizing effects of PARPis.

Another typical feature of cancer is the loss of contact inhibition. In cultures, nontransformed cells cease to proliferate upon reaching confluence. This process is accompanied by downregulation of PARP1 expression via transcription factors, specifically factors 1 and 3 (Sp1 and Sp3), as demonstrated in various primary cells [53] and in keratinocytes [54]. This may be related to intrinsic cell cycle-related gene regulation [55,56] and/or integrin signaling [57]. How PARP1 expression changes when neoplastic cells are in contact with each other (e.g., in 2D or 3D cell culture models or in vivo tumors) and how adhesion factors in the tumor stroma affect PARP1 expression are largely unexplored.

### 2.2. Resisting Cell Death

Neoplastic cells rewire prosurvival and prodeath pathways, resulting in resistance to cell death [13,14]. Several PARP enzyme functions are linked to this cancer hallmark. As DNA damage sensor proteins, PARP1 and PARP2 contribute to DNA repair and, as such, they are bona fide prosurvival factors in DNA damage scenarios. In fact, the use of PARPis in BRCA-deficient ovarian or breast carcinomas takes advantage of this synthetic lethal effect (synthetic lethality is a situation where a defect in one gene/protein is not lethal for the cell, but when combined with another gene/protein defect it turns lethal) of PARylation inhibition (blocking DNA single-strand break repair) in BRCA mutant tumors, which are deficient in homologous recombination repairing [11,58]. Even before the discovery of the synthetic lethality-based effects of PARPis, extensive preclinical investigations proved that PARPis sensitize cancer cells to several chemotherapeutic drugs, ionizing radiation, and phototherapy [59,60,61,62,63]. Since the molecular background and clinical implications of PARPi therapy have been extensively reviewed [10,12,19,64], we will focus on less-known connections between cancer cell death sensitivity/resistance and the PARP enzyme family.

Some chemotherapeutic approaches are mediated, rather than counteracted, by *PARP-1* activation. An example is the killing of nonsmall cell lung cancer (NSCLC) cells by radiosensitization and the chemotherapeutic naphtoquinone drug, β-lapachone. This therapeutic regime induces reactive oxygen species (ROS) production via redox cycling of the drug, resulting in DNA damage and excessive activation of PARP1 [65]. PARP-1-mediated NSCLC cell death displays features of both necroptosis and parthanatos [66]. Thus, the extensive literature about the cytotoxicity of uncontrolled PARP1 activation (classical and noncanonical parthanatos) in oxidative stress-related pathologies contributes to our understanding of the role of PARP1-mediated cell death in anticancer treatment modalities [9,67,68].

Intracellular localization of PARP1 greatly affects its cell death resistance function. Although the enzyme predominantly localizes in the nucleus, PARP1 has also been detected in the cytoplasm, such as in the hydroquinone-induced, ROS-dependent death of TK6 human lymphoblastoid cells [69]. Interestingly, cytoplasmic, but not nuclear, PARP1 causes the resistance of pancreatic cancer cells to apoptosis induced by monoclonal antibodies to death receptor type 5 (DR5). DR5 is the receptor for the death ligand TRAIL (tumor necrosis factor-related apoptosis-inducing ligand) [70]. Cytoplasmic interaction of *PARP-1* and the death-inducing signaling complex (DISC) may be important to the cell death regulatory role of PARP1 [70] (Figure 1). The mechanism of PARP1 translocation and generalization of this phenomenon require further investigation.

Co-activation or stimulation of cell survival signaling pathways (e.g., NFκB and Wnt-β-catenin) by PARP1 [71,72] may also contribute to cell death resistance [73,74]. On the other hand, PARP1 is a negative regulator of the prosurvival PI3K-Akt pathway (Figure 1). This latter connection is important for the tissue-saving effects of PARPis in ischemia-reperfusion models [75,76] and may also underlie cancer cell resistance to PARPis [77] or chemotherapeutics, such as paclitaxel [78]. Along these same lines, the PARPi olaparib enhances the activation of the antioxidant master regulator transcription factor nuclear factor (erythroid-derived 2)-like 2 (NRF2) [79]. In nontransformed tissues, this is clearly a prosurvival effect. However, redox regulation has a controversial role in cancer [80]. Therefore, the importance of the PARP1-NRF2 axis in cancer awaits confirmation. 

The multilevel connections between PARP1 and p53 were discussed in detail in previous sections. In addition to stimulating cell cycle arrest and senescence, p53 also triggers apoptosis by inducing the expression of p53 upregulated modulator of apoptosis (PUMA) and phorbol-12-myristate-13-acetate-induced protein 1 (NOXA) (Figure 1). These BH3-only proteins interact with and switch off the antiapoptotic effects of Bcl-2, unleashing the mitochondrial apoptotic signaling pathway. The mutual interdependence of *PARP-1* and p53 (see Section 1 and Section 2) affect DNA damage-induced and p53-dependent apoptosis [22] and PARP-1-mediated necrotic cancer cell death [81]. Considering the stimulus- and cell type-dependent effects of p53 activation, ranging from enhanced proliferation to cell death, the net result of p53-PARP1 interactions in various tumor treatment regimens need to be reviewed individually. 

As a crucial mechanism of cellular homeostasis, autophagy plays a role in oncogenesis suppression [82]. In established tumors, autophagy is considered to be a survival-assisting process that is stimulated not only by tumor cell starvation but also by chemotherapy. Therefore, inhibition of the autophagy pathway may enhance the effects of antitumor interventions [82]. The role of PARP1 in cancer cell autophagy is somewhat controversial. On the one hand, PARP1 may mediate autophagy (such as in nasopharyngeal carcinoma cells) [83]. On the other hand, the PARP inhibitor olaparib has also induced autophagy in BRCA mutant breast cancer cells. Under nutrient deprivation, ROS production and DNA damage trigger PARP1 activation [84]. A key molecular event underlying the autophagy-promoting role of PARP1 is the formation of PARP1/AMP-activated protein kinase (AMPK, an energy-sensor protein involved in the initiation of autophagy) complexes; PARylation and nucleocytoplasmic translocation of AMPKs initiates the autophagy process [85]. The other PARP1 substrates involved in this process remain to be revealed in targeted proteomic screens. The combination of PARPi therapy with autophagy inhibition may also act synergistically, as demonstrated with the PARPi niraparib in laryngeal squamous carcinoma cells [86].

A key mechanism by which tumor cells evade the toxic effects of chemotherapeutics is the activity of ABC (ATP-binding cassette) transporters. The PARPi veliparib sensitizes liver cancer cells to doxorubicin. Doxorubicin accumulates in veliparib-treated cells by inhibiting the ATPase activity of ABCB1 (also known as P-glycoprotein) without affecting the expression level of the transporter [87]. The important question raised by this study is whether this is due to an off-target effect of the drug or due to PARP inhibition. In *PARP-1* deficient cells, ABCB1 was upregulated, rendering cells resistant to doxorubicin [88]. Similarly, after treatments with PARPis, several leukemic cell lines became more resistant to anticancer agents, including both DNA-damaging and nondamaging drugs [89]. Since resistance to antiFAS was also increased by the PARP inhibitors, resistance is likely due to interference with apoptotic signaling rather than upregulation of multidrug resistance. In summary, the majority of studies suggest increased drug resistance upon PARP inhibition, which should be considered if PARPis will later be used in combination with traditional chemotherapeutic drugs.

Information on the role of other PARPs in cell death resistance is scarce. Somewhat surprisingly, studies linking PARP2 with cell death resistance are lacking. The overlapping roles of PARP1 and PARP2 in DNA damage response (DDR) and the fact that most PARPis target both enzymes suggest that PARP2 also contributes to PARPi-induced cancer cell death. In a noncancer-related setting, PARP2 contributed to a key step (apoptosis inducing factor (AIF) translocation) in PARylation-dependent cell death [90]. Moreover, the metabolic and autophagy-promoting roles of PARP2 [91,92] are likely to affect cancer cell sensitivity to DNA-damaging agents. Similar to PARP2, not much is known about the potential role of PARP3 in cancer cell death resistance apart from a study that reported poor prognosis of patients with PARP3 overexpressing breast cancers who received chemotherapy. PARP13 plays a role in sensitizing cells to TRAIL-induced apoptosis. Similar to its role in antiviral defense where it mediates degradation of viral RNA, PARP13 also binds to several mRNAs, resulting in their destabilization. PARP13 targets the mRNA of the proapoptotic cytokine TRAILR4 (a decoy receptor for TRAIL). Moreover, the ER protein PARP16 participates in the ER stress response, a signaling network that can also determine cell fate [93]. Considering the common occurrence of ER homeostasis disturbances, including protein folding problems in tumors [94], one can speculate that PARP16 may affect cancer cell response to treatment.

### 2.3. Enabling Replicative Immortality

Two barriers can curb the proliferative lifespan of normal human cells: replicative senescence and a state of severe genomic instability called crisis [95]. Both stages are initiated at the telomeres, which progressively shorten with each round of cell division due to the end-replication problem of linear DNA. This eventually triggers replicative senescence, a practically irreversible arrest of proliferation sustained by a resistance to mitogenic signals and an inability to re-enter the cell cycle. If a cell still manages to bypass senescence, telomere erosion and deprotection exposes the chromosome ends. The deprotected chromosome ends are attended to by double-strand break (DSB) repair, leading to end-to-end fusions, dicentric chromosomes, nondisjunction events, and breakage–fusion–bridge cycles. The resolution of these aberrations scrambles the karyotype, threatening cell viability. The DDR is co-opted as the core signaling route for replicative senescence, whereas the cell cycle arrest is orchestrated by a network of cyclin-dependent kinase inhibitors (CKI) and the p53 and Rb signaling pathways [96].

Human telomeres consist of several kilobase pairs of double-stranded TTAGGG repeats and 50–400 nucleotides of a 3′-OH single-stranded overhang of the same sequence. The telomeres are associated with a six-subunit telomere-specific protein complex called shelterin [97]. Three subunits of shelterin, telomeric repeat-binding factors 1 and 2 (TRF1, TRF2), and protection of telomeres 1 (POT1), bind the TTAGGG repeats directly. The other three subunits, TRF1-interacting nuclear factor 2 (TIN2), TPP1, and repressor/activator site-binding protein homolog (RAP1), are involved in averting DNA repair mechanisms from processing the chromosome ends as DNA damage sites and in telomerase regulation. The t-loop, a lariat structure formed by the 3′ overhang invading the double-stranded telomeric DNA, serves to bury the free DNA end.

In cancer cells, two telomere maintenance mechanisms can be reactivated to maintain telomeric DNA at lengths sufficient to protect the cells from senescence [98]. Although telomerase activity is virtually absent from normal human nonstem adult somatic cells, in cancer, activating mutations in the promoter region, promoter methylation, and gene copy number amplification of human telomerase reverse transcriptase (hTERT), the catalytic subunits of telomerase are among the most frequent genetic/epigenetic alterations [99]. In addition, 5–15% of cancers express undetectable levels of hTERT and instead regenerate their telomeres via a recombination-directed mechanism called the alternative lengthening of telomeres (ALT) [100,101]. 

*PARP-1* is detected sporadically in normal telomeres but accumulates on telomeres affected by DNA damage or erosion due to telomerase deficiency [102] (Figure 2). *PARP-1* is involved in the normal repair of intratelomeric single- and double-strand breaks (SSBs and DSBs) [103]. *PARP-1* can also be inadvertently activated by the double- to single-strand transition segment at the t-loop base unless the transition segment is masked and protected from unwinding by TRF2. If activated by binding to the three-way junction at the base of the t-loop, *PARP-1* facilitates the recruitment of Holliday junction resolvases, promoting t-loop cleavage and eliciting a response similar to intratelomeric DSBs [104,105,106]. *PARP-1* activation in either case leads to (alt)-nonhomologous end joining (alt-NHEJ). TRF2 safeguards the telomere by inhibiting the ataxia-teleangiectasia mutated (ATM) signaling cascade downstream from the DSB recognition. To dispense with this roadblock to DNA repair, *PARP-1* PARylates TRF2, diminishing its DNA-binding activity [102]. Noncovalent binding of PAR by TRF2 has a similar effect [107]. The binding of *PARP-1* to telomeres is also counteracted by TIN2 [105]. TRF2 and TIN2 block maximal *PARP-1* activation at the telomere cooperatively, suggesting that they act through independent mechanisms [105]. The telomeric guanine-rich 3′ single-stranded overhang can adopt the G-quadruplex (G4) fold, a noncanonical nucleic acid structure that antagonizes telomerase. G4-stabilizing small molecular ligands induce the removal of TRF2 and POT1 from telomeres, causing t-loop instability and bridge–fusion events [108,109]. As a therapeutically actionable possibility, concomitant silencing or inhibition of *PARP-1* prevents the repair of G4 stabilization-induced DNA breaks, leading to increased chromosome abnormalities and inhibition of cell growth [110]. 

Activated by strand breaks, *PARP-2* may also mediate DNA repair at telomeres. Like *PARP-1*, *PARP-2* PARylates TRF2. PARylation causes TRF2 to dissociate from DNA, facilitating DNA access for the repair machinery [107]. Telomere erosion and anaphase fusion bridges occur at higher frequencies in PARP-2^−/−^ mouse cells, supporting a role for *PARP-2* in the maintenance of telomere integrity [107,111]. *PARP-2* also regulates recombination-driven telomere regeneration in ALT [107]. PARG depletion and consequent PAR accumulation protect cells from spontaneous telomere recombination or fusion during or after telomere replication in S or G2, which is consistent with the overall protective effect of PAR on telomere integrity [112].

The other mechanism related to how PARPs influence telomere stability is the regulation of telomerase. *PARP-1* silencing in human squamous epithelial carcinoma cell lines and mouse embryonic stem cells revealed that *PARP-1* is responsible for recruiting KLF4 to the *hTERT* promoter to maintain telomerase expression [113]. In PARP-1^−/−^ mice, telomeres are substantially shortened and exhibit spontaneous abnormalities [114,115]. *PARP-1* modulates telomerase activity via two mechanisms: regulation of telomerase-associated protein 1 (TEP1), a subunit of the telomerase holoenzyme, and PARylation of hTERT [116,117]. TNKS1 (PARP5a, ARTD5) and TNKS2 (PARP5b/ARTD6) regulate telomere cohesion and length through TRF1 [118,119,120,121,122]. TRF1 impedes the access of telomerase to telomeres. PARylation of TRF1 by TNKS1/2 results in TRF detachment and proteasomal degradation of TRF1, enabling telomere elongation [118,123]. Accordingly, TNKS1 overexpression increases telomere length, whereas TNKS1 depletion or inhibition shortens telomeres [124]. TIN2 binds to both TRF1 and TNKS1 to prevent the PARylation of TRF1 by TNKS1 [125]. Telomerase activity in some cancers is upregulated through the downregulation of TRF1, TRF2, or TIN2 gene expression [126]. PARP4 binds to TEP1, a telomerase subunit and a minor protein component of cytoplasmic vault particles, but to date there are no data to explain whether this affects telomere regulation [127]. TNKS1/2 and “dual” PARP1/PARP2/TNKS1/TNKS2 inhibitors are promising targets for telomere-targeted chemotherapy [128]. Coinhibition of TNKS1 and telomerase synergistically shorten telomere length in gastric cancer cells [129]. Inhibitors of the telomere-associated PARPs block TRF2 release from telomeres and the combined inhibition of telomerase and PARPs accelerates telomere shortening in fibrosarcoma cells [130]. 

In contrast, *PARP-3* and telomerase expression negatively correlate in NSCLC and in various cancer cell lines [131,132]. *PARP-3* inhibition is a cancer therapy target based on its involvement in mitosis progression that also depends on telomere integrity [133]. *PARP-3* inhibition increases telomerase activity, which can be beneficial in treating cancers like NSCLC where telomere attrition has been documented to be associated with a poorer prognosis due to a higher incidence of chromosomal rearrangements [134]. 

### 2.4. Genome Instability and Mutation

Cancer relies on the failure of mechanisms designed to conserve the sequence and organization of genetic material, which is referred to as “genome instability” or “hypermutability” [135]. The evolutionary model of tumor progression posits that enhanced accumulation of genotypic alterations is guided by neoDarwinian principles via a process of diversification and selection. Changes stimulating proliferation or resilience in the face of stress confer survival advantage for cells, leading to the expansion and dominance of that cell lineage [136,137]. One class of cancer-susceptible genes does not directly propel proliferation but instead guards the integrity of the genome. These genes are classically called “genome caretakers” and anomalies in these genes are among the most frequent causes of inherited predisposition to cancer [138]. PARPs carry many functions that align them with the definition of “genome caretakers”.

#### PARPs in DNA Repair

DNA repair is the swift identification and correction of DNA damage due to environmental and endogenous factors or oncogenic mutations. DNA repair coincides with a pause in replication and transcription through a sustained lesion. PARPs have been implicated in base excision and single-strand break repairs (BER and SSBR), in double-strand break repairs (DSBR) by both homologous recombination (HR) and nonhomologous end joining (NHEJ), and in nucleotide excision repairs (NER) (Figure 2A–C). In contrast to the rest of the PARP family, *PARP-1*, *PARP-2*, and *PARP-3* have DNA-binding capabilities. These PARPs localize to DNA damage and this association enhances their basal catalytic activity. *PARP-1* can recognize single-strand breaks, double-strand breaks, nicks, and non-B DNA structures (such as DNA hairpins, crosses, and loops) [8]. *PARP-2* and *PARP-3* are selectively activated by DNA breaks carrying a 5’ phosphate; these PARPs presumably respond to ligation-competent intermediates later in the repair process [139]. Other PARPs, such as tankyrases, may also regulate DDR by interacting with lesion-associated proteins.

- DSBR

One of the first critical decisions made at break ends is the choice between resection or conversion of the DNA ends into single-stranded overhangs. This decision directs the process towards NHEJ or HR and is crucial in determining the degree of sequence corruption. *PARP-1*, *PARP-2*, and *PARP-3* are instrumental in the DSBR pathway selection (Figure 2A). *PARP-1* protects the HR pathway from Ku70/80 complex interference by temporarily impeding Ku70/80 from binding to the DSB. PARP1 is also necessary for preparing the neighboring chromatin before Ku arrives [140,141,142,143]. *PARP-1* antagonizes DNA end resection by occluding the broken end and blocking the MRN–RPA–BLM–EXO1 and the MRN–RPA–BLM–DNA2 exonuclease complexes from loading onto the DNA. PARP inhibition leads to hyper-resected DSB ends [141]. Interestingly, these complexes rely on the PAR binding of MRE11 [144]. PARP1 may also stimulate the PAR-dependent recruitment of the tumor protein p53-binding protein 1 (53BP)- replication timing regulatory factor 1 (RIF1) complex, a DNA resection suppressor, and the RNA-binding nonPou domain-containing octamer-binding protein (NONO) that also stimulates NHEJ and represses HR [145,146]. *PARP-1* interacts with and PARylates BRCA1, a governing factor of HR; these actions stabilize the association of BRCA1 with the receptor-associated protein-80 kD (RAP80) complex, repressing recombination events by restricting end resection [147]. Defective PARylation of BRCA1 gives rise to uncontrolled HR and genome instability, resulting in genomic abnormalities similar to those observed in the absence of BRCA1 in some breast cancers [148]. 

PARP3 affects NHEJ at a later step by retaining the X-ray repair cross-complementing 4 (XRCC-4)/DNA ligase IV complex at the DSB site [149,150] (Figure 2A). In contrast to *PARP-1* and 3, *PARP-2* facilitates DNA resection, favoring HR, single strand annealing (SSA), or altNHEJ [141,151]. TNKS1/2 interact with the mediator of DNA damage checkpoint protein 1 (MDC1), a protein involved in HR and NHEJ; these proteins are recruited to DSB sites [152]. MDC1 facilitates the deposition of histone post-translational modification marks, which lead to BRCA1 recruitment. TNKS1/2 also stabilize the BRCA1 complex through the recruitment of mediators of RAP80 interactions and targeting of 40-kD proteins (MERIT40). Loss of TNKS function interferes with HR, probably via effects on BRCA1 [152].

- NER

*PARP-1* is promptly activated by UV-C- and UV-B-induced thymine dimers [153]. *PARP-1* associates with, is stimulated by, and PARylates damaged DNA-binding protein 2 (DDB2). In addition, *PARP-1* directs XPC to the lesion, improving the effectiveness of global genomic NER [154,155] (Figure 2C). PARP1 and the NER scaffold protein XPA mutually regulate each other’s functions. Despite the fact that PAR binding diminishes the direct DNA binding affinity of XPA, PAR formation around the lesion is necessary for the early recruitment of XPA. The PAR-bound XPA in turn strongly enhances the activity of PARP1 [156,157]. PARP1 also interacts directly with the ssDNA binding protein RPA, which stabilizes the NER bubble. When bound to ssDNA, RPA inhibits PARP1 and increases its turnover on the DNA [158]. These molecular events are believed to fine-tune the assembly and/or disassembly of the NER complex [157]. On the whole, the efficient removal of UV-induced photolesions requires *PARP-1*, which is consistent with the observation that impaired *PARP-1* function increases UV-induced skin cancer in mice [154,159].

- SSBR and BER

PARylated *PARP-1* or other proteins serve as a scaffold on which X-ray repair cross-complementing 1 (XRCC-1), DNA polymerase β, DNA ligase 3 (LIG3), and polynucleotide kinase 3-prime phosphatase (PNKP) interact to execute the SSBR process [160]. *PARP-1* PARylates tyrosyl-DNA phosphodiesterase 1 (TDP1), enhancing TDP1 recruitment to TOP1 cleavage complexes to release trapped abortive TOP1. The resulting nick is repaired by SSBR [161]. Both *PARP-1* and PARG were critical for the rapid global rates of SSBR, whereas *PARP-2* depletion had only minor effects on SSBR [162]. Cell-free extracts from PARP-1^−/−^ mice repaired apurinic sites inefficiently [163]. *PARP-1* has a high affinity for 5′-dRP-containing nicked intermediates [164]. Of the proteins involved in the BER pathway, *PARP-2* interacts with *PARP-1*, XRCC1, DNA polymerase β, and LIG3. Like *PARP-1*, *PARP-2* also PARylates XRCC1. PARP1 differentially regulates the short- and long-patch (SP and LP) BER pathways. The interaction of PARP1 with the nicked intermediate interferes with the access of both flap structure-specific endonuclease 1 (FEN1) and Polβ. The autoPARylation of PARP1 disinhibits strand displacement synthesis by Polβ and 5′-flap cleavage by FEN1 in LP-BER [165,166] (Figure 2B). *PARP-2*-deficient mouse embryonic fibroblast cells displayed a delay in DNA strand break repair similar to *PARP-1* deficient cells [167]. *PARP-1* blockades are synthetically lethal in XRCC1-deficient sporadic ovarian carcinoma and ductal breast carcinoma [168,169]. However, PARP1 and PARP2 still regulate two different yet connected aspects of DNA base damage tolerance. Both promote BER, but *PARP-2* is required to stabilize replication forks that encounter BER intermediates [170]. In response to oxidative DNA damage, TNKS1 is recruited to telomeres through TRF1. TNKS1 inhibition abolishes the accumulation of XRCC1 and POLβ at telomeric DNA damage sites. Thus, TNKS1 facilitates SSBR specifically at damaged telomeres through PARylation of TRF1 [171].

Understanding the exact mechanism by which PARPs are involved in DDR will help identify genetic markers for PARPi therapy. Moreover, insight into the molecular mechanisms of the synthetic lethality between PARPs and various repair factors may broaden our therapeutic arsenal against cancer. 

### 2.5. Reprogramming Energy Metabolism

**Figure 3:**
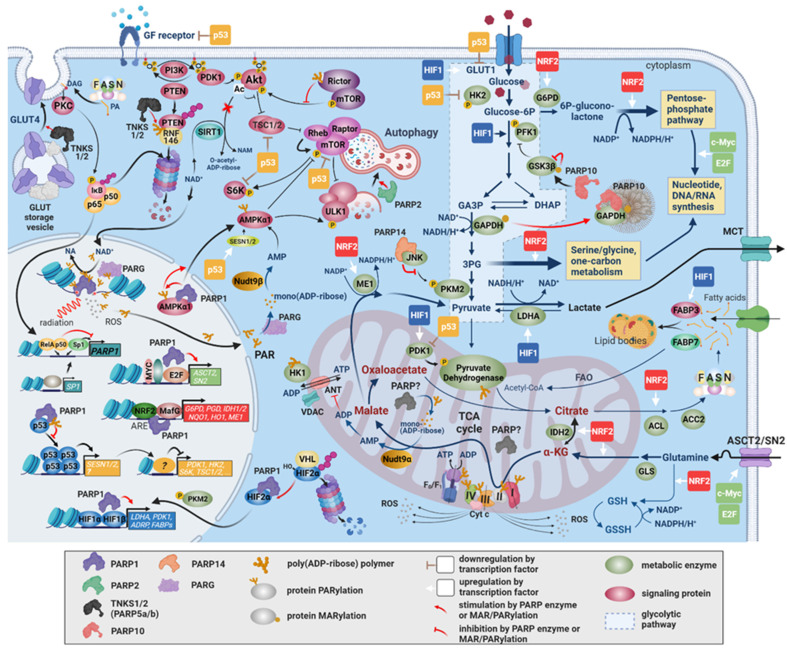
The involvement of PARPs in metabolic reprogramming in cancer. The rewired metabolism in cancer is established by oncogenic signaling and transcriptional programs switched on in response to changes in the internal milieu of cancer cells and the tumor microenvironment. PARPs exert a multifaceted influence on metabolism either through stimulating these transcriptional programs or via direct PAR/MARylation of metabolic proteins. By enhancing HIF1/2 target gene expression through HIF stabilization and coactivation, *PARP-1* and *PARP-2* increase glycolytic flux at the levels of glucose entry into the cell and into the glycolytic pathway and at the level of pyruvate removal by *LDHA*. *PARP-1* induces c-MYC and E2F expressions and is a coactivator of NRF2. NRF2, c-MYC, and E2F responsive genes include enzymes in the pentose phosphate, serine/glycine, and C1-metabolic pathways, which channel away glycolytic intermediates for the synthesis of nucleotides, nucleic acids, and NADPH that are required for biomass production. Inhibition of GSK3β and GAPDH by PARP10 and of PKM2 by PARP14 also divert intermediates to anabolic pathways branching off from glycolysis. The uptake of metabolic precursors, such as glucose, glutamine, and fatty acids, are stimulated by PARP1/2-enhanced HIF1/2, c-Myc, and E2F-dependent expressions of their respective transporters. NRF2 enhances glutamine metabolism, glutathione synthesis and reduction, and NADPH-producing enzyme expression (*ME1*, *IDH2*) for elevated oxidative stress tolerance. This leads to anaplerotic recircuiting of the TCA cycle. A major factor in the downregulation of mitochondrial function and oxidative phosphorylation is the inactivation of the pyruvate dehydrogenase complex by PDK1, which is also a target gene of HIF1/2. The electron transport chain complexes may be inhibited by PARylation, although the identity(ies) of the mitochondrial PARP(s) is/are uncertain. PARP1, PARP2, and PARP10 expressions may also negatively influence mitochondrial biogenesis and performance through SIRT1 and PGC1α. The PI3K/Akt-pathway is a major stimulator of cell survival and growth through glucose transporter expression, protein synthesis, and the protection of mitochondria. Under pronounced genotoxic stress, the consumption of cytoplasmic NAD^+^ by activated *PARP-1* antagonizes the pathway due to the inhibition of NAD^+^-dependent deacetylation of Akt by SIRT1. Additionally, Rictor, the regulatory subunit of mTORC2, is an activator of Akt. Rictor is inhibited by noncovalently bound PAR. The inhibition of the PI3K-Akt-mTOR axis, the activation of AMPK, and the activation of the HIF transcriptional response are all mechanisms through which *PARP-1* supports nutrient acquisition by autophagy, another feature of tumor metabolism. Functional p53 in cancers exerts a negative regulatory effect on the expression of several GFR–PI3K–Akt–mTOR pathway components, glycolytic flux determining enzymes, and PDK1, which may be derepressed by *PARP-1*. Fatty acids produced in cancer cells by upregulated FASN expression induce *PARP-1* gene expression through the downregulation of NFκB and the upregulation of Sp1, buttressing the tumor cell’s tolerance of DNA damage.

A recent surge of interest in prospective metabolism-normalizing therapeutic strategies has advanced our understanding of metabolic reprogramming in cancer well beyond the Warburg effect. Tumor cell metabolism has its own list of hallmarks. Briefly, cancer cells (i) have enhanced deregulated glucose, glutamine, fatty acid, and cholesterol uptake; (ii) channel glycolysis/TCA-cycle intermediates to biosynthetic pathways; (iii) upregulate de novo lipogenesis, storage, and β-oxidation to generate ATP; (iv) accumulate anabolic precursors by alternative modes of nutrient acquisition; (v) produce oncometabolites, which affect their epigenetic gene regulation; and (vi) thrive off specific effects these metabolic products have on their environment [180]. PARPs have a profound and manifold influence on these aspects of metabolism (Figure 3). The influence of PARPs on tumor metabolism is exercised through transcriptional mechanisms, direct PAR/MARylation of metabolic proteins, detached PAR chains, or indirectly through changes in NAD^+^ and ATP levels. PARPs directly regulate enzymes or control key metabolic regulatory factors. 

HIFs orchestrate a transcriptional program that controls angiogenesis, matrix remodeling, cell death, metastasis formation, metabolism, and growth in response to hypoxia in cancer [181,182,183,184,185]. PARP1 physically interacts with HIF-1α and HIF2 and protects them from von Hippel–Lindau tumor suppressor-mediated ubiquitylation and degradation. In addition, PARP1 functions as a transcriptional coactivator for HIF-1α and HIF2 [186,187]. HIF signaling drives glycolysis by inducing *hexokinase 2* (*HK2*) and *phosphofructokinase 1* (*PFK1*), reduces flux through the TCA cycle, and diminishes mitochondrial oxygen consumption through the induction of *lactate dehydrogenase A* (*LDHA*) and pyruvate dehydrogenase kinase 1 (PDK1)-mediated inhibition of the pyruvate dehydrogenase complex (PDC). Glyceraldehyde-3-phosphate dehydrogenase (GAPDH), one of over two dozen proteins in central metabolic pathways that are mono or poly ADP-ribosylated, is inhibited by PARP-1-mediated PARylation [188,189,190,191,192]. *PARP-10* physically interacts with MARylates and recruits GAPDH to cytosolic membraneless granules [193]. Glycogen synthase kinase 3β (GSK3β) is inhibited by PARP-10-dependent MARylation, which promotes glycolytic and anabolic pathways [188]. Interaction between c-Jun N-terminal kinase 1 (JNK1) and PARP14 prevents JNK1 activation of pyruvate kinase muscle-type 2 (PKM2) and PKM2 nuclear translocation, where PKM2 would enhance glycolytic gene expression through HIF1α and MYC [194]. Prevention of glycolytic gene expression slows glycolysis, channeling intermediates into the pentose phosphate (PP) and serine/glycine synthetic pathways [195]. Hexokinase 1 (HK1), which is localized to the outer surface of the mitochondria, is inhibited by PAR chains released from the nucleus upon genotoxic stress [196]. 

*PARP-1* antagonizes the activity of the PI3K/Akt pathway; the PI3K/Akt pathway enhances glycolytic flux through glucose transposters (GLUTs), HK, and PFK2, and promotes nucleotide, protein, and lipid biosynthesis and autophagy in cancer [76,197]. Acetylation by p300 and PCAF blocks the binding of Akt and phosphoinositide-dependent kinase-1 to phosphatidylinositol (3,4,5)-trisphosphate [198]. *PARP-1* inhibits the protein deacetylase sirtuin 1 (SIRT1), leaving Akt acetylated, which prevents Akt from phosphorylating its target, the mechanistic target of rapamycin complex 1 (mTORC1) [199]. Akt needs to be phosphorylated for full activation by mTORC2. Rictor, the regulatory subunit of mTORC2, is inhibited by noncovalently bound PAR [190,200]. PARylation of the tumor suppressor PTEN by TNKS1/2, however, promotes its degradation and advances tumor growth [201]. On a cautionary note, therapeutic *PARP-1* inhibition can lead to PI3K/Akt stimulation, which may contribute to therapy resistance [78]. 

NRF2 governs the expression of genes involved in most redox-balancing antioxidant and xenobiotic elimination systems, including glutathione synthesis, ROS elimination, NADPH synthesis, xenobiotic metabolism, and drug excretion [202]. During oxidative stress, NRF2 is stabilized by the suspension of ubiquitylation and proteasomal removal. NRF2 enters the nucleus dimerizes with a small Maf protein and activates over 200 genes with antioxidant response elements (ARE). This antioxidant response allows cancer cells to tolerate higher ROS production, survive radiotherapy and chemotherapy, and metastasize [203,204,205,206]. Accordingly, increased NRF2 expression is frequently found in human cancer [207,208,209]. NRF2 redirects glucose and glutamine into anabolic pathways during metabolic reprogramming [210]. *PARP-1* interacts with MafG and ARE to stimulate the NRF2 transcriptional response [211]. By inducing *glucose-6-phosphate dehydrogenase* (*G6PD*), *phospho-gluconate dehydrogenase* (*PGD*), *transketolase* (*TKT*), and transaldolase 1 (*TALDO1*), NRF2 diverts glucose toward NADPH and nucleotide synthesis through the PPP. By upregulating *malic enzyme 1* (*ME1*), NRF2 short-circuits the TCA cycle and supplies more NADPH for glutathione reduction [210,212]. 

*PARP-1* forms a nuclear complex with AMPKα1 that is disrupted when AMPK is PARylated. PARylated AMPK translocates to the cytosol where it is activated by elevated AMP levels produced from the degradation of PAR chains by PARG and nucleoside diphosphates linked to x (NUDIX) hydrolases. Active AMPK phosphorylates unc 51-like autophagy, activating kinase 1 (ULK1), and inactivates mTORC1 and p70S6 kinase (p70S6K), stimulating autophagy [85]. The released AMP also leads to competitive inhibition of the mitochondrial adenine nucleotide translocator (ANT), linking DNA-dependent PARPs to retrograde inhibition of oxidative phosphorylation, the electron transport chain, and the TCA-cycle [213]. 

Tumor cells upregulate glucose transporters to ensure the constant availability of glucose for their ramped-up metabolism [214]. Oncogenic mutations in the PI3K/Akt, mTOR, HIF, Ras, or p53 signaling pathways involved in the regulation of GLUT function render glucose acquisition in cancer cells independent of external stimuli [215,216]. Increased GLUT1 expression is a feature of many malignancies and GLUT1 levels correlate with high-grade tumors [217,218,219,220]. *PARP-1* antagonizes Akt, which is crucial for both the expression and translocation of GLUT1 to the plasma membrane [76,77,221,222]. *PARP-1* silencing decreases HIF1-mediated transcriptional activation and GLUT1 expression in chronic myelogenous leukemia cells [186]. Certain types of breast cancer and multiple myeloma are GLUT4-dependent [223,224]. GLUT4 plasma membrane exposure is also primarily regulated by PI3K/Akt in response to insulin and IGF. PARP inhibition or TNKS1 knockdown is associated with downregulation of GLUT4 and GLUT4 storage vesicle proteins, resulting in impaired stimulated GLUT4 translocation to the plasma membrane [225,226]. 

The inhibition of *PARP-1* enhances mitochondrial biogenesis and metabolism through SIRT1-dependent gene regulation [227]. During oxidative stress, *PARP-1* silencing in lung adenocarcinoma cells increases basal oxidative phosphorylation and the mitochondrial reserve capacity and prevents mitochondrial dysfunction. This suggests that *PARP-1* is an important regulator of mitochondrial function and cellular bioenergetics, not only when overactivated by DNA-damaging stressful signals, but also in unchallenged conditions [228]. PARP inhibition reproduces the same effects, implying that the enzymatic activity of *PARP-1* (or a PARP) rather than physical interactions of the protein is required. Functional *PARP-1* deficiency results in a marked and selective increase in the mitochondrial NAD^+^ pool, which increases conversion of NAD^+^ to NADH in the TCA cycle, an increasing electron flow, and elevated mitochondrial respiration. The evidence for mitochondrial PARPs remains equivocal [229,230,231]. However, intramitochondrial PARylation was reported upon oxidative or nitrosative stress or excitotoxicity in cultured cortical neurons and isolated mitochondria [185]. Subunits of all mitochondrial electron transport chain complexes and several subunits of the ATP synthase are targets for PARylation. As for the identity of the mitochondrial ADP-ribosylating enzyme, cytosolic *PARP-1* may translocate into the mitochondria by an unresolved mechanism involving interaction with mitofilin [232]. 

*PARP-10* silencing in several cancer cell lines induces mitochondrial oxidative metabolism and upregulated AMPK activity [233]. The role of MARylation in this process is unknown. *PARP-10* expression correlates inversely with the expression of PGC-1α, a driver of mitochondrial biogenesis and fatty acid oxidation.

Cancer cells upregulate plasma membrane and cytosolic fatty acid transporters, such as CD36, fatty acid transport protein (FATP), membrane-associated fatty acid binding protein (FABPpm), and fatty acid binding proteins (FABPs), to meet the increased demand for phospholipids. Silencing of these transporters was sufficient to mitigate tumor growth [234]. HIF1 upregulates FABP3 and FABP7 to stimulate cytosolic fatty acid transport and lipid accumulation.

The β-oxidation of fatty acids (FAO) is emerging as a drugable pathway in cancers that rely on FAO for stemness, proliferation, drug resistance, or metastasis [235]. The most prominent transcriptional regulators of FAO are MYC, JAK/STAT3, and the peroxisome proliferator-activated receptors (PPARs) [235]. PARPs may counteract FAO through several mechanisms. PPARα PARylation by *PARP-1* suppresses FAO [236]. Malonyl-CoA is an inhibitor of fatty acid synthesis. Acetyl-CoA carboxylase 2 (ACC2), which is responsible for malonyl-CoA production, is repressed in many neoplastic cells via sirtuin-mediated histone deacetylation. NAD^+^ consumption by activated PARPs inactivates sirtuins and promotes FAO through peroxisome proliferator-activated receptor gamma coactivator 1-alpha (PGC1α), PPARα, and ACC2 [237]. HIF-1 inhibits the medium- and long-chain acyl-CoA dehydrogenases (MCAD and LCAD), resulting in decreased ROS levels and enhanced proliferation. Fatty acid synthase (FASN) is often overexpressed in human cancer and is associated with increased resistance to chemo- or radiotherapy [238]. Palmitate, the catalytic product of FASN, downregulates NF-κB and increases SP1 expression. These two effects derepress and induce the *PARP-1* gene, respectively. Elevated *PARP-1* expression enhances NHEJ, resulting in higher genotoxin resistance [239]. Patients with FASN overexpressing tumors, therefore, may benefit from combined therapies that include PARPi.

Several additional metabolic roles assigned to PARPs (e.g., autophagy, nucleotide biosynthesis, SIRT signaling) cannot be discussed here due to space limitation. See Figure 3 and cited references.

## 3. Conclusions

The roles of PARP enzymes in various HoCs as discussed in this paper and in the companion paper must be viewed in their complexities [18]. For example, the metabolic and proliferation-promoting roles of PARPs affect cancer cell sensitivity to chemo- and radiotherapy [240]. Investigating the effects of PARPi therapy in a certain type of tumor at a higher level of complexity (considering synergistic or possible antagonistic effects of drug combinations and the parallel activation of pro-death and cytoprotective mechanisms) requires systems biology approaches [241]. Similarly, a systematic analysis is clearly needed to investigate the potential of combination therapies involving PARPis and inhibition of survival mechanisms. PARPis were initially developed with the goal of suppressing DNA damage repair and achieving a high level of replication stress that would eventually engage the same cell death pathways that are activated in response to DNA damage-inducing cytotoxic chemotherapies [242]. A large fraction of combinational therapies in the preclinical or clinical stages pursue the improvement of treatment response and the avoidance of the development of resistance by combining PARPis with these cytotoxic drugs. Combinations of PARPis with talozolomide, platinum-based compounds, topoisomerase inhibitors, and base analogs are found in numerous clinical trials [242]. A growing number of new approaches pair PARPis with agents targeted at specific molecular alterations in tumors. These molecularly targeted therapies include RTK inhibitors, checkpoint kinase 1/2 (CHK1/2) inhibitors, ATR inhibitors, Wee1 inhibition, PI3K inhibitors, HDAC inhibitors, IGFR inhibitors, Raf inhibitors, MEK inhibitors, or drugs interfering with sex hormone synthesis [242,243,244,245]. The special edge of PARPis was derived from the discovery of synthetic lethality. Most of these agents generate homologous recombination deficiency, even in HR-proficient tumors where the conditions for synthetic lethality are not given, mostly by downregulating BRCA1/2, Rad51, or other HR factors [242,246]. Besides these strategies, which still aim at accumulating DNA damage, other novel combination therapies leverage less traditional PARP effects, such as one that counteracts the adverse proliferative transcriptional effects of PARPis [247]. As highlighted in this paper, various cellular effects of “minor” PARP enzymes await further exploration as these are also possible anticancer targets. The most promising such explorations may focus on telomerase regulation by TNKS1, TNKS2, and PARP3, and the role of PARP16 in the ER stress response. The possible roles of NAD^+^ synthesis and PAR-degrading enzymes have not yet been investigated as possible targets in cancer even though the effects of PARP1 and PARG inhibition often result in similar rather than opposite outcomes in DNA damage scenarios [248,249]. Promising preclinical data also support the targetability of NAD synthesis enzymes [250]. Moreover, the role of PARPs in the regulation of cancer cell sensitivity to cytotoxic immune cells (CTLs, NK cells, and CAR-T cells) also awaits investigation [18].

## Supplementary Materials

The following are available online at https://www.mdpi.com/article/10.3390/cancers13092042/s1, Figure S1: Nomenclature, schematic domain architecture and enzymatic activities of the human PARP family members, Figure S2: Reaction mechanism of the generation and removal of mono- and poly(ADP-ribose), Table S1: PARP inhibitors approved for cancer therapy and in clinical trial phase.
