# Supplementary material for: The PARP Enzyme Family and the Hallmarks of Cancer Part 1. Cell Intrinsic Hallmarks
Source: Cancers (Basel). 2021 Apr 23;13(9):2042. doi: 10.3390/cancers13092042 (PMC8122967; doi:10.3390/cancers13092042)
Supplement: Supplementary file 1 [file cancers-13-02042-s001.zip › cancers-1160576-supplementary.pdf]

PARP inhibitors presently approved by the FDA for cancer therapy

Olaparib

4-[[3-[4-(cyclopropanecarbonyl)piperazine-1-carbonyl]-4-fluorophenyl]methyl]-2H-phthalazin-1-one

Sold under the trade name Lynparza. It is used for advanced ovarian cancer that has been treated with three or more lines of chemotherapy and HER2 negative+BRCA negative breast cancer after the failure of other lines of chemotherapy.[a]

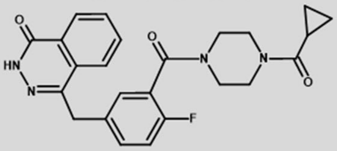

Rucaparib

6-fluoro-2-[4-(methylaminomethyl)phenyl]-3,10-diazatricyclo[6.4.1.$0^{4,13}$]trideca-1,4,6,8(13)-tetraen-9-one

The drug, Rubraca is used since 2016 in selected BRCA-mutated ovarian cancer patients after at least two lines of platinum-based therapy. Also effective against ovarian tumors with preserved BRCA1/2 but homologous recombination deficiency and loss of heterozygosity.[b]

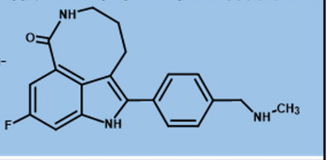

Niraparib

2-[4-[(3S)-piperidin-3-yl]phenyl]indazole-7-carboxamide

Approved by the FDA in March 2017 for maintainance therapy in ovarian cancer. Sold under the trade name Zejula.[c]

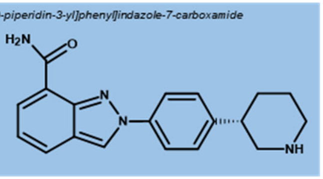

Talazoparib

(11S,12R)-7-fluoro-11-(4-fluorophenyl)-12-(2-methyl-1,2,4-triazol-3-yl)-2,3,10-triazatricyclo-[7.3.1.$0^{5,13}$]trideca-1,5(13),6,8-tetraen-4-one

Approved by the FDA in 2018 for BRCA-mutated and HER2 negative locally advanced or metastatic breast cancer. Sold under the trade name Talzenna.[d]

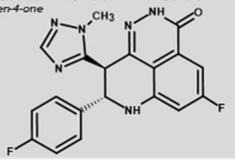

New PARP inhibitors in the clinical trial stage against cancer

Senaparib (IMP-4297)

5-fluoro-1-({4-fluoro-3-[4-(pyrimidin-2-yl)piperazine-1-carbonyl]-phenyl}methyl)quinazoline-2,4(1H,3H)-dione

In Phase I trial for advanced solid tumors (prostate, breast and pancreatic cancer). In Phase II against BRCA1/2 mutated ovarian cancer with at least 2 prior lines of standard systemic therapy. In Phase III as maintainance in ovarian cancer.[e]

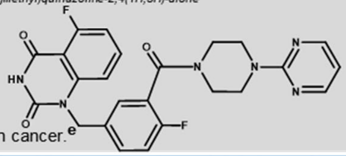

Veliparib (ABT-888)

2-[(2R)-2-methylpyrrolidin-2-yl]-1H-benzimidazole-4-carboxamide

Currently in over two dozen mostly Phase I/II trials for various solid tumors, breast, serous, fallopian tube cancer, glioma or leukemia in monotherapy or in combination with platinum, temozolomide, ATR, CDK, tyrosine kinase inhibitors, immune checkpoint inhibitors or radiotherapy.[f]

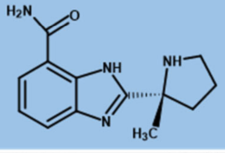

Fluzoparib (SHR-3162)

4-[[4-fluoro-3-[2-(trifluoromethyl)-6,8-dihydro-5H-[1,2,4]triazolo-[1,5-a]pyrazine-7-carbonyl]phenyl]methyl]-2H-phthalazin-1-one

In >15 Phase I-III clinical trails in monotherapy or in combination with temozolomid or apatinib in breast, renal, ovarian, pancreatic peritoneal, fallopian tube and small cell lung cancer.[g]

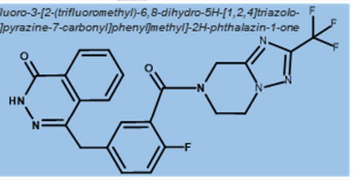

Pamiparib (BGB-290)

(2R)-14-fluoro-2-methyl-6,9,10,19-tetrazapentacyclo[14.2.1.$0^{2,6}$.$0^{8,18}$.$0^{12,17}$]-nonadeca-1(18),8,12(17),13,15-pentaen-11-one

Currently evaluated in high grade serous ovarian cancer or carcinosarcoma that has progressed on PgP substrate chemotherapy or PARPi. In Pase II in combination with temozolomide in hereditery leiomyomatosis and renal cell cancer.[h]

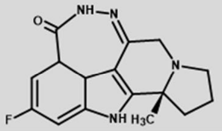

Stenoparib (2X-121; E7449)

8-[(1,3-dihydro-2H-isoindol-2-yl)methyl]-1,2-dihydro-3H-pyridazino[3,4,5-de]quinazolin-3-one

PARP1/2 inhibitor also inhibiting TNKS1/2. Currently in Phase II trials in patients with metastatic breast or advanced ovarian cancer selected based on BRCA status.[i]

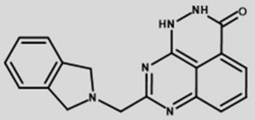

Venadaparib (IDX-1197)

4-[[3-[3-[(cyclopropylamino)methyl]azetidine-1-carbonyl]-4-fluorophenyl]methyl]-2H-phthalazin-1-one

In Phase I/II trials in patients with Homologous recombination repair mutated solid tumors and in Phase I trial in combination with irinotecan or capecitabine+oxaliplatin in advanced gastric cancer.

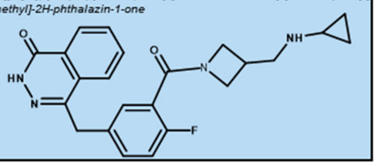

Supplemantary Table S1. PARP inhibitors approved for cancer therapy and in clinical trial phase.

[a] [1,2]

[b] [1,2]

[c] [1,2]

[d] [1,2]

[e] NCT04584515, NCT04434482, NCT04089189, NCT04351165, NCT04169997, NCT04822961

[f] [1,3], NCT02158507, NCT02723864, NCT01434316, NCT03061188, NCT01351909, NCT02595905, NCT03581292, NCT01009788, NCT02470585, NCT02163694, NCT01139970, NCT00588991, NCT01489865, NCT01366144, NCT01149083, NCT01386385, NCT01012817, NCT01585805, NCT03227016, NCT02152982, NCT03400306, NCT00576654, NCT02890355, NCT00740805, NCT01711541, NCT01618357, NCT00989651, NCT02921256

[g] [1,4], NCT04659785, NCT03945604, NCT04517357, NCT04425876, NCT04718740, NCT04535687, NCT04694365, NCT03509636, NCT03075462, NCT03863860, NCT04296370, NCT04228601, NCT04300114, NCT04229615, NCT04041011, NCT04400188, NCT04341077, NCT04782089

[h] [1,5], NCT03933761, NCT04614909, NCT04603365, NCT04796454, NCT03333915, NCT03994211, NCT03150862, NCT03427814, NCT03150810, NCT03519230, NCT03749187, NCT03575065, NCT03914742

[i] [1,6], NCT03878849, NCT03562832

[j] [1,7], NCT03317743, NCT04174716, NCT04725994

ATR: ataxia teleangiectasia related; CDK: cyclin-dependent kinase, PgP: P-glycoprotein

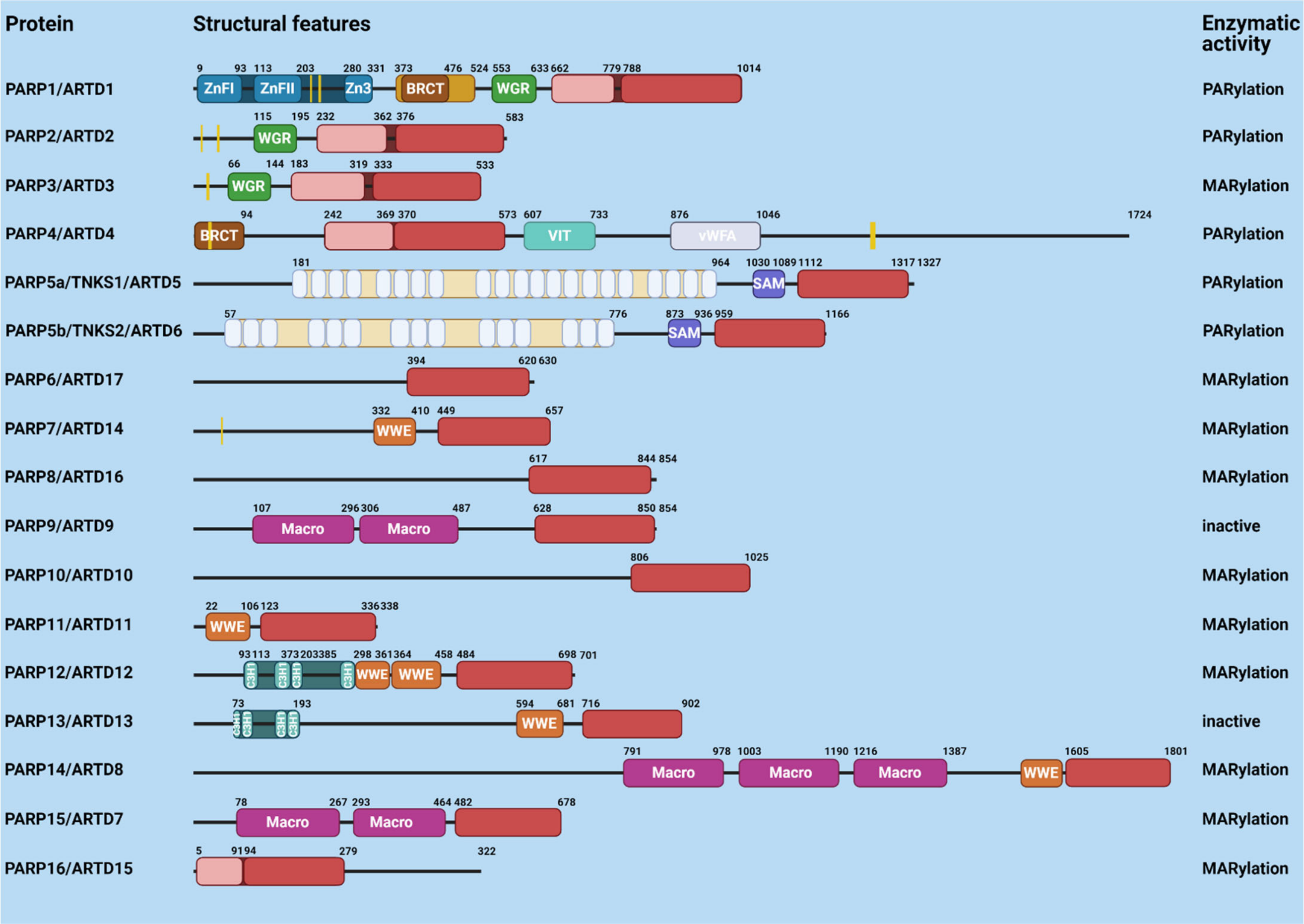

Supplementary Figure S1. Nomenclature, schematic domain architecture and enzymatic activities of the human PARP family members. The conserved domains are shown based on the Prosite domain and functional site database. PARP catalytic domain (maroon); alpha helical subdomain (pink) [*PS51060*]; ADP-ribosyl transferase subdomain (red) [*PS51059*]; WGR (bright green): W/G/R motif containing domain; BRCT (brown): breast cancer type 1 susceptibility protein carboxy-terminal domain [*PS50172*]; automodification region (ochre); WWE (orange): conserved W, W and E residues containing protein domain [*PS50918*]; SAM (violet): sterile alpha motif domain [*PS50105*]; ankyrin repeat motifs (white); ankyrin repeat domain (beige) [*PS50297*]; VIT (bluish green): vault protein inter-alpha-trypsin domain [*PS51468*]; vWFA (grey): von Willebrand factor type A domain [*PS50234*]; ZF-I and ZF-II (blue): PARP-type zinc finger domain [*PS50064*]; Zn3 (blue): third PARP-1 zinc-binding domain [8]; PARP1 DNA-binding domain (dark blue); C3H1: (turquoise): Cys/Cys/Cys/His zinc finger motif [*PS50103*]; C3H1 RNA-binding domain (dark green); Macro (magenta): macrodomain [*PS51154*]; NLS (yellow): nuclear localization signal

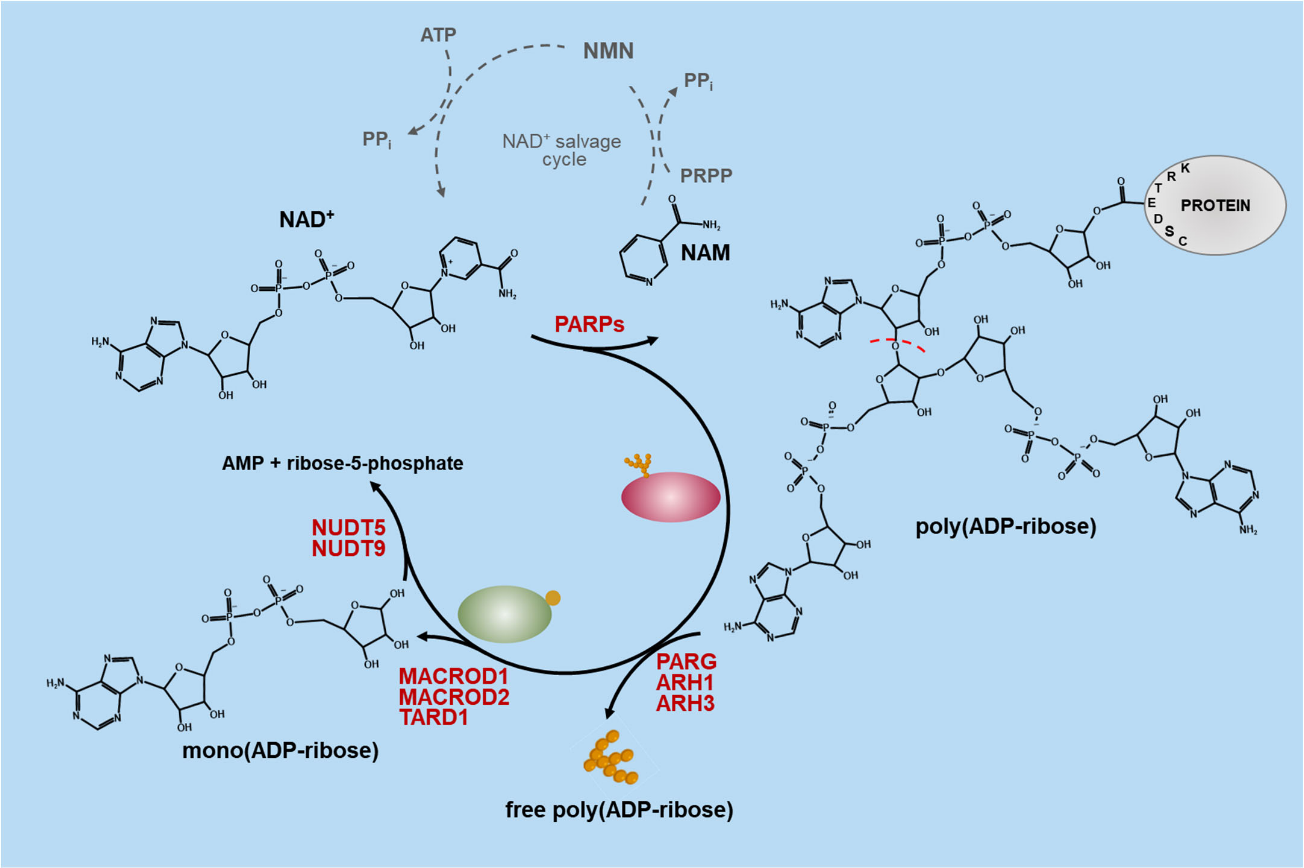

Supplementary Figure S2. Reaction mechanism of the generation and removal of mono- or poly(ADP-ribose). PARPs use NAD^+ to mono- or poly(ADP-ribosylate) proteins on glutamate, aspartate, lysine, arginine, serine, threonine or cystein residues. The reaction releases nicotinamide (NAM) that is converted back to NAD^+ in the posphatidyl-ribosyl-pyrophosphate (PRPP) and ATP-consuming NAD-salvage cycle. Poly(ADP-ribose) chains are linear or branching. PARG, ADP-ribosylhydrolase 1 or 3 (ARH1 or 3), acting both as endo- and exo-glycohydrolases, cleave protein bound poly(ADP-ribose) releasing freely diffusible polymers and depolymerize these chains to mono(ADP-ribose) units [9]. Protein-attached mono(ADP-ribose) or the last unit of a protein bound PAR chain is removed by the MacroD1, MacroD2 or terminal ADP-ribosyl glycohydrolase (TARD1) proteins [10]. Free mono(ADP-ribose) is hydrolysed by members of the nucleoside diphosphate linked moiety X (NUDIX) hydrolase family, NUDT5 and NUDT9 to ribose- phosphate and AMP [11].

1. Curtin, N.J.; Szabo, C. Poly(ADP-ribose) polymerase inhibition: past, present and future. *Nat Rev Drug Discov* **2020**, *19*, 711-736, doi:10.1038/s41573-020-0076-6.
2. Yi, M.; Dong, B.; Qin, S.; Chu, Q.; Wu, K.; Luo, S. Advances and perspectives of PARP inhibitors. *Exp Hematol Oncol* **2019**, *8*, 29, doi:10.1186/s40164-019-0154-9.
3. Donawho, C.K.; Luo, Y.; Luo, Y.; Penning, T.D.; Bauch, J.L.; Bouska, J.J.; Bontcheva-Diaz, V.D.; Cox, B.F.; DeWeese, T.L.; Dillehay, L.E., et al. ABT-888, an orally active poly(ADP-ribose) polymerase inhibitor that potentiates DNA-damaging agents in preclinical tumor models. *Clin Cancer Res* **2007**, *13*, 2728-2737, doi:10.1158/1078-0432.Ccr-06-3039.
4. Wang, L.; Yang, C.; Xie, C.; Jiang, J.; Gao, M.; Fu, L.; Li, Y.; Bao, X.; Fu, H.; Lou, L. Pharmacologic characterization of fluzoparib, a novel poly(ADP-ribose) polymerase inhibitor undergoing clinical trials. *Cancer Sci* **2019**, *110*, 1064-1075, doi:10.1111/cas.13947.
5. Wang, H.; Ren, B.; Liu, Y.; Jiang, B.; Guo, Y.; Wei, M.; Luo, L.; Kuang, X.; Qiu, M.; Lv, L., et al. Discovery of Pamiparib (BGB-290), a Potent and Selective Poly (ADP-ribose) Polymerase (PARP) Inhibitor in Clinical Development. *J Med Chem* **2020**, *63*, 15541-15563, doi:10.1021/acs.jmedchem.0c01346.
6. McGonigle, S.; Chen, Z.; Wu, J.; Chang, P.; Kolber-Simonds, D.; Ackermann, K.; Twine, N.C.; Shie, J.L.; Miu, J.T.; Huang, K.C., et al. E7449: A dual inhibitor of PARP1/2 and tankyrase1/2 inhibits growth of DNA repair deficient tumors and antagonizes Wnt signaling. *Oncotarget* **2015**, *6*, 41307-41323, doi:10.18632/oncotarget.5846.
7. Lee, M.; Park, J.-T.; Yang, J.-h.; Kim, D.; Je, I.-G.; Lee, Y.S.; Jeong, J.; Song, D.K.; Park, S.; Lee, H.-S., et al. Abstract A106: Development of IDX-1197, a novel, selective, and highly potent PARP inhibitor. *Molecular Cancer Therapeutics* **2018**, *17*, A106-A106, doi:10.1158/1535-7163.Targ-17-a106.
8. Langelier, M.F.; Servent, K.M.; Rogers, E.E.; Pascal, J.M. A third zinc-binding domain of human poly(ADP-ribose) polymerase-1 coordinates DNA-dependent enzyme activation. *J Biol Chem* **2008**, *283*, 4105-4114, doi:10.1074/jbc.M708558200.
9. Rack, J.G.M.; Ariza, A.; Drown, B.S.; Henfrey, C.; Bartlett, E.; Shirai, T.; Hergenrother, P.J.; Ahel, I. (ADP-ribosyl)hydrolases: Structural Basis for Differential Substrate Recognition and Inhibition. *Cell Chem Biol* **2018**, *25*, 1533-1546.e1512, doi:10.1016/j.chembiol.2018.11.001.
10. Feijs, K.L.H.; Cooper, C.D.O.; Žaja, R. The Controversial Roles of ADP-Ribosyl Hydrolases MACROD1, MACROD2 and TARG1 in Carcinogenesis. *Cancers (Basel)* **2020**, *12*, doi:10.3390/cancers12030604.
11. Formentini, L.; Macchiarulo, A.; Cipriani, G.; Camaioni, E.; Rapizzi, E.; Pellicciari, R.; Moroni, F.; Chiarugi, A. Poly(ADP-ribose) catabolism triggers AMP-dependent mitochondrial energy failure. *J Biol Chem* **2009**, *284*, 17668-17676, doi:10.1074/jbc.M109.002931.
